# Supplementary material for: Content-rich biological network constructed by mining PubMed abstracts
Source: BMC Bioinformatics. 2004 Oct 8;5:147. doi: 10.1186/1471-2105-5-147 (PMC528731; doi:10.1186/1471-2105-5-147)
Supplement: Additional File 2 — The original results of the above study (non-essential files are deleted to keep the file size under the limit set by BMC bioinformatics). [file 1471-2105-5-147-S2.bz2 › chilibotAdditionalFile2/dip05/100ID7759493E427/html/P_NP.html]

 


 **P** and **NP** 
  
Found 2734 abstracts in PubMed, retrieved 05.  
 

 What does Google say? 
 PDF only 
| .edu only 

---

**Interactive relationship** (e.g. stimulation, inhibition, etc)

**Neutral relationship**- **P**  rats were found to have significantly greater brain damage, compared with  **NP**  rats, in the posterior perirhinal and posterior entorhinal cortexes, 239% 50%  **p**  <.  Ref: 12878913 Alcohol Clin Exp Res, 2003
- Phospho extracellular signal regulated kinase immunohistochemistry stained prominently in damaged brain areas.CONCLUSIONS The  **P**  rat line, a genetic model of alcoholism, shows greater region specific brain damage due to binge ethanol treatment than its genetic counterpart, the  **NP**  rat line.  Ref: 12878913 Alcohol Clin Exp Res, 2003

**Non-interactive relationship** (e.g. studied together, co-existance, homology, etc.)

- To this aim, rats of the sNP and Sardinian alcohol preferring sP lines were exposed to a 108 day sucrose fading procedure, which was virtually identical to that previously used with  **NP**  and  **P**  rats.  Ref: 12878272 Alcohol, 2003
- Binge Ethanol Treatment Causes Greater Brain Damage in Alcohol Preferring  **P**  Rats Than in Alcohol Nonpreferring  **NP**  Rats.  Ref: 12878913 Alcohol Clin Exp Res, 2003
- Covalent attachment of 2C5 antibody was performed via a micelle incorporated PEG PE with the distal terminus of the PEG block activated with  **p**  nitrophenylcarbonyl group pNP  [ **NP** ]  PEG PE.  Ref: 12881194 J Drug Target, 2003
- Animals were killed, transcardially perfused, and fixed, and their brains were removed, sectioned, and stained by using the amino cupric silver stain of de Olmos or by using immunohistochemistry for phospho extracellular signal regulated kinases and other antigens.RESULTS Significant brain damage was found in the olfactory bulbs, posterior perirhinal cortex, and entorhinal cortex in both  **P**  and  **NP**  rats.  Ref: 12878913 Alcohol Clin Exp Res, 2003
- To investigate the role of genetics in binge drinking induced brain damage, we studied bidirectionally selected rat lines, the alcohol preferring  **P**  and the alcohol nonpreferring  **NP**  rat lines.METHOD  **P**  and  **NP**  rats were treated with a 4 day binge ethanol protocol.  Ref: 12878913 Alcohol Clin Exp Res, 2003
